# Comprehensive Update on Keloid Management

**DOI:** 10.1055/a-2698-3574

**Published:** 2026-01-30

**Authors:** Michael H. Tirgan, Qi Yin, Albert Wolkerstorfer, Barbu Gociman, Tae Hwan Park

**Affiliations:** 1 Keloid Research Foundation, New York, New York, United States; 2 Department of Dermatology, Amsterdam UMC Location University of Amsterdam, Amsterdam, The Netherlands; 3 Division of Plastic and Reconstructive Surgery, Department of Surgery, University of Utah School of Medicine, Salt Lake City, Utah, United States

**Keywords:** keloid, fibroblast, chemotherapy, cryotherapy, radiation therapy

## Abstract

Keloids remain one of the most challenging conditions in cutaneous wound healing, marked by complex pathophysiology and notoriously high recurrence rates. Although a universally accepted standard of care is still lacking, recent advances have significantly improved our understanding and management of this fibrotic disorder. Emerging evidence highlights genetic predisposition, prolonged inflammatory response, and aberrant fibroblast activity as key contributors to keloid formation. Current therapeutic approaches focus on multimodal strategies, including intralesional corticosteroids, cryotherapy, radiation therapy, and chemotherapy. Intralesional triamcinolone remains a first-line treatment, while chemotherapy agents like 5-fluorouracil and vincristine have shown promising efficacy in refractory cases. Surgical excision, often combined with adjuvant therapies such as radiation, is considered for large or recurrent lesions. Given the high recurrence rate, patient-centered, evidence-based treatment algorithms are essential. Stratifying keloids into categories enables tailored interventions. Both established and emerging treatments are now evolving toward more personalized and less invasive approaches to improve outcomes and patient satisfaction. Multimodal, individualized treatment approaches—guided by lesion morphology, anatomical location, treatment history, and patient factors—are essential for optimizing outcomes. Emerging therapies are expanding the therapeutic arsenal, offering additional strategies for resistant or recurrent cases. Moreover, the integration of molecular and genetic insights is paving the way for the development of targeted therapies, which may ultimately transform keloid treatment into a more precise and effective discipline. Future studies should focus on large-scale trials to establish standardized, data-driven treatment guidelines for keloids.

## Introduction

Keloids are characterized by genetic and molecular abnormalities that interfere with normal wound-healing processes. Although the exact mechanisms remain incompletely understood, current research highlights that the pathogenesis involves dysregulation in one or more phases of wound healing, including inflammation, proliferation, and remodeling. 1 This dysregulation appears to be driven by a complex interplay of genetic predisposition, vascular dysfunction, inflammation, and fibrosis.

Emerging evidence suggests that individuals with keloids have a heightened and prolonged inflammatory response to skin injury, which may be accompanied by abnormal fibroblast activity, excessive collagen deposition, and aberrant angiogenesis. Despite these insights, the intricate molecular and cellular mechanisms underlying keloid formation are yet to be fully elucidated, representing a significant challenge for researchers and clinicians alike.

Clinically, keloids are abnormal fibrotic growths that arise from improper wound healing in susceptible individuals. These lesions typically develop after skin injuries such as trauma, surgery, burns, acne, or even minor inflammations like insect bites or ear piercings. Unlike hypertrophic scars, which remain confined to the original wound boundary, keloids extend beyond the original wound boundary, often continuing to grow for months or years. Keloids are often associated with pain, pruritus, disfigurement, and functional impairment, significantly impacting a patient's quality of life.

In addition to their physical and psychological burden, keloids are notoriously resistant to treatment, with a high propensity for recurrence, especially after surgical excision. This challenges clinicians to develop and adopt multimodal treatment approaches tailored to the individual needs of each patient.

In this review, we will explore the current understanding of keloid pathogenesis, highlighting advancements in genetic and molecular research, and discuss evidence-based strategies for the clinical management of keloids. By bridging the gap between basic science and clinical practice, we aim to provide insights into more effective and personalized approaches to treating keloids.

## Treatment Strategy

Keloid treatment should be personalized, multimodal, and patient-centered. Factoring in age, prior treatments, social circumstances, and psychological impact allows for better outcomes and improved patient satisfaction. 2 A combination of minimally invasive treatments, with targeted escalation if needed, provides the best balance between efficacy and recurrence prevention for patients with early-stage keloids.

One of the biggest challenges in keloid treatment is the lack of data-driven approaches due to the paucity of well-conducted clinical trials. Despite the abundance of keloid patients worldwide, there is no universally accepted treatment protocol, primarily because

1. Heterogeneity of keloids: Keloids vary in size, location, patient demographics, genetic predisposition, and response to treatments, making standardization difficult.
2. Lack of large-scale, high-quality clinical trials: Most existing studies are small, retrospective, or lack control groups, leading to inconsistent findings.
3. Historical reliance on empirical treatment: Many treatments (steroids, cryotherapy, surgery) have been expert-driven or based on anecdotal evidence rather than robust clinical data.

Treatment plans listed below are guided by the appearance and, to some extent, the location of the keloid lesions. When deciding on a treatment choice, one can categorize keloid lesions into four general categories.

- Very early-stage disease (papular/linear keloids)
- Nodular/Tumoral keloids
- Flat keloids/keloid patches
- Very large keloids

### Algorithm to Treat Keloid Papules

All early-stage keloid papules shall first be treated with intralesional triamcinolone (ILT). If the lesions respond to the first injection of ILT, the same treatment shall be repeated once every 3 to 4 weeks until maximum response is achieved.

If the lesion does not respond to the first injection of ILT, a second injection of ILT will be attempted. Lack of response to two ILT injections or the growth of keloid after the first ILT injection is grounds for switching the treatment. Repeated ILT injections when the intervention is ineffective are a known risk factor for the worsening of keloid lesions. The following line of treatment for all such lesions will be intralesional chemotherapy (ILC). If ILC fails, the following treatment modality will be contact cryotherapy.

### Treatment of Nodular/Tumoral Keloids

Most tumoral and bulky keloid lesions can be successfully debulked using cryotherapy. Once the mass of a bulky keloid is reduced to the skin level, attention must be directed to controlling the remnant of the keloid tissue that may persist at the base of the keloid. ILC shall be used to treat any such remnant.

### Treatment of Very Large Keloids

Treating very large keloids is challenging. Quite often, these patients have undergone prior keloid removal surgeries. For these patients, a surgical approach followed by an adjuvant treatment, either radiation therapy or ILC, should be considered. This will be further discussed later in the surgical therapy section.

## Management of Keloids

### Cryotherapy

The use of cryotherapy in the treatment of keloids dates back to 1950, 3 with several authors confirming the efficacy of this treatment modality. 4 5 Proper patient selection is crucial. Cryotherapy can be effective for nodular keloids, particularly those on the ear. M.H.T. prefers contact cryotherapy with swabs dipped in liquid nitrogen, which are then applied directly to the keloid tissue. Cryotherapy for large lesions can be a painful procedure, making pain control critical. After cryotherapy, the treated tissue becomes edematous, necrotic, and may ooze for several days until a dark scab forms on the surface of the treated area. This scab remains for several weeks before falling off. To achieve the desired results, cryotherapy must be repeated every 6 to 8 weeks.

### Corticosteroids

Intralesional corticosteroids are the most commonly used non-surgical keloid treatment. Corticosteroids are frequently used in keloid treatment and can be administered topically, with or without occlusion, or intralesionally using conventional (hypodermic) needles or jet injectors. Recently, laser-assisted drug delivery (LADD) using ablative fractional lasers has also been proposed for delivering medication in keloids. Intralesional corticosteroid administration (ICA) is a first-line treatment for keloids, with triamcinolone being the most commonly used. 6 Reports indicate that intralesional corticosteroids as monotherapy for keloids result in a 50 to 100% improvement in both objective and subjective symptoms, 6 with a recurrence rate of 20 to 37%, 7 8 rising to 50% after 5 years. 9 Common adverse events associated with intralesional corticosteroids include atrophy (5–75%) and telangiectasia (10–80%). 10

The treatment outcomes of ICA may be affected by various operator-dependent factors, such as the type, volume, and concentration of corticosteroid; the number and interval of treatment sessions; the size of the needle and syringe; and the techniques involved in manual injection. Currently, there is considerable heterogeneity and incomplete reporting concerning many aspects of ICA in randomized controlled trials, as shown in a recent scoping review. 11 Additionally, a recent survey indicates a significant variation in the practice of ICA. 12 A recent e-Delphi study confirmed consensus on several aspects of ICA, including treatment goals, indications for corticosteroid use, triamcinolone acetonide (TAC) 40 mg/mL as the preferred corticosteroid administered at a maximum of 80 mg per month and at 4-week intervals, minimizing pain during administration, the use of 1-mL syringes and 25 or 27 gauge needles, blanching as the endpoint of successful infiltration, the caution against subcutaneous injection, and the option to create needle perforations in very firm keloids prior to infiltration. However, consensus was not achieved regarding TAC dosing, methods of prior local anesthesia, and injection locations. 12

Studies on the mechanisms of corticosteroids in keloids are still limited. It is believed that corticosteroids promote keloid regression through various mechanisms, 6 including effects on inflammation, fibroblast proliferation, collagen production and breakdown, and apoptosis. Direct anti-inflammatory effects have been assumed to be the primary mechanism of action for corticosteroids in keloids. However, the specific anti-inflammatory effects induced by corticosteroids in keloids remain unclear. In a study involving 17 patients with keloids, biopsies were taken before and after treatment with TAC injections given every 2 weeks for a total of three injections over 6 weeks. Immunohistochemical analyses showed no significant decrease in the anti-inflammatory IL-6 levels. 13 14 However, an increase in MMP-13 ( *p* < 0.05) was observed, which is an enzyme responsible for breaking down extracellular matrix components, including collagen. Additionally, a decrease in fibronectin ( *p* < 0.001) was noted; this glycoprotein of the extracellular matrix is often elevated in keloids. Moreover, a significant decrease in collagen type III ( *p* < 0.05) was observed, while the decrease in collagen type I was not statistically significant. Notably, fibrogenic transforming growth factor (TGF)-β levels did not significantly decline after treatment in this study. This finding contrasts with another study that demonstrated that 20 μM TAC reduced the production of fibrogenic TGF-β1 by both normal and keloid fibroblasts.

### Intralesional Chemotherapy ( Fig. 1 )

**Fig. 1:**
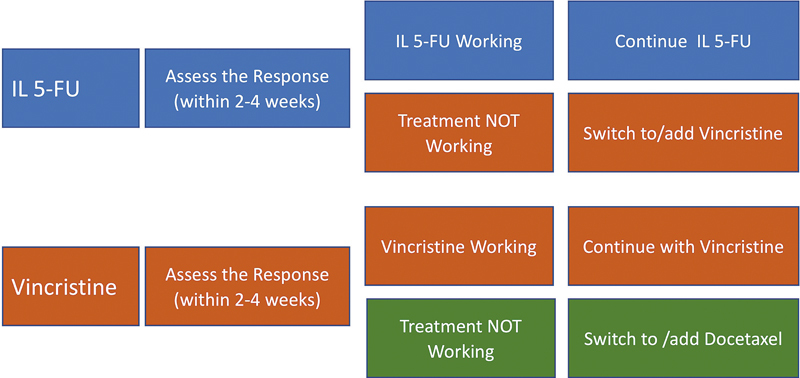
The treatment flowchart for intralesional chemotherapy for keloids. 5-FU, 5-fluorouracil.

Fitzpatrick first introduced ILC using 5-fluorouracil (5-FU) to treat keloid lesions in 1989. 15 In the United States, and perhaps globally, 5-FU is the most commonly used chemotherapy drug.

ILC is most appropriate for treating papular, linear, or flat keloids. It should be used only as a second or later line of treatment after documenting previous treatment failure with such as ILT. ILC is generally not recommended as a first-line treatment in a general setting. 8

In choosing ILC, the treating physician must obtain proper treatment history from the patient and affirmatively establish that ILT has already been used, at least twice, and that the keloid has either failed to respond or has progressed with ILT.

Upon initiation of ILC, the injected lesion(s) must be inspected within 3 to 4 weeks, at which point the patient and the provider will together determine whether ILC was effective. If ILC is determined to have been effective, it can be repeated during the follow-up session. Injections should be repeated every 3 to 4 weeks until the maximum response is achieved, at which point the treatment shall be halted and the lesions shall be photographed and observed.

In contrast to ILT, ILC can be a curative treatment. New and small keloid lesions may totally disappear after one or two ILC injections. Lesions that respond to ILC should be monitored for potential recurrence.

In patients with multiple lesions, some will respond better than others. Once a lesion shows an adequate response, it should no longer be treated. Patients should be advised to monitor their lesions and return if a recurrence occurs.

In choosing chemotherapy drugs for treatment of a benign disease, only drugs that are non-carcinogenic, non-mutagenic, and with a reasonable short- and long-term safety profile should be used. 16 Among the numerous available chemotherapeutic drugs, the author recommends the following three drugs for intralesional treatment of keloids 8 : Most commonly, three drugs (5-FU, vincristine, and docetaxel) must be used in sequence. 5-FU inhibits deoxyribonucleic acid (DNA) synthesis by way of inhibition of thymidylate synthase, targeting rapidly dividing cells. Vincristine binds to β-tubulin and inhibits microtubule polymerization. 17 18 Docetaxel also binds to β-tubulin within microtubules; however, it promotes polymerization and prevents depolymerization of microtubules, thereby stabilizing microtubules excessively, making them non-functional. 19

The International Agency for Research on Cancer (IARC) and the U.S. National Toxicology Program (NTP) do not list 5-FU, vincristine, or docetaxel as a known or suspected carcinogen. Although bleomycin has been reported to be effective in the treatment of keloids, the drug is a known carcinogen. The NTP lists bleomycin as “reasonably anticipated to be a human carcinogen” based on sufficient evidence of carcinogenicity in experimental animals and supporting genotoxicity data. Similarly, the IARC has classified related antitumor antibiotics as Group 2B carcinogens (possibly carcinogenic to humans), and bleomycin is included among genotoxic antineoplastic agents that warrant caution. Therefore, the authors advise against using bleomycin in the treatment of keloid patients, especially given its long-term carcinogenic potential.

5-FU should be used first, and if the keloid lesions show resistance to this drug, vincristine should be used. Docetaxel should only be used in lesions that have failed to respond to both 5-FU and vincristine. This sequence is derived from safety, side effect profiles, and the complexities involved in the proper administration of each drug.

We previously published detailed protocols outlining the dosage schedules, administration techniques, and sequencing strategies for the chemotherapeutic agents used in the intralesional treatment of keloidal lesions.

#### Recommended Dosage

In preparing vincristine for intralesional injections, the author recommends a very low dose of this drug, no more than 20 μg of vincristine, to be added to a standard preparation of triamcinolone for ILT injection, with a final volume of 0.4 mL of the solution that is to be used for injection.

Extreme care must be given to choose and prepare the correct dosage of vincristine in the smallest possible volume to minimize the chance of causing damage from vincristine to the normal surrounding tissues. Vincristine is a very potent vesicant, and if injected, it is commercially available in 1- to 2-mL vials at a concentration of 1 mg/mL. This concentration is equivalent to 1,000 μg of the drug per mL. Extreme care must be exercised to extract the correct amount of the drug into the syringe that is to be used for intralesional vincristine injection. The dose and volume of vincristine to be injected into the keloid lesion depend on the size of the lesion.

#### Recommended Dosage for Docetaxel

Docetaxel is available in 1- or 4-mL vials at a concentration of 20 mg/mL. In preparing docetaxel for ILC injections, M.H.T. recommends a very low dose of this drug to be added to the standard preparation of triamcinolone for ILT injection, with no more than 400 μg of docetaxel per 1.0 mL of the final preparation that is to be injected.

#### Preparation of the Solution for Injection

Premedication is recommended for all patients undergoing intralesional docetaxel injection to prevent allergic reactions.

The author uses the following steps in preparing intralesional docetaxel.

1. Docetaxel is first drawn into a 1-mL syringe to the level of the first graduated line on the syringe. Each graduated line represents a volume of 2/100 mL and will contain 400 μg of docetaxel.
2. Triamcinolone is then added in the same syringe by drawing 2/10 mL of Kenalog-10, which contains 2 mg of triamcinolone (see KRF Guideline - ILT).
3. Normal saline is then drawn into the same syringe to a total volume of 1.0 mL.

This mixture will contain 400 μg of docetaxel and 2 mg of triamcinolone. Each graduated line of the mixture will contain 8 μg of docetaxel and is ready for injection into the target keloid lesions. The amount of docetaxel that is to be injected into the keloid lesion depends on the size of the lesion.

#### Preparation of the 5-Fluorouracil Solution for Injection

5-FU is commercially available in 10-mL or larger-size vials at a standard concentration of 50 mg/mL. The author uses the following steps in preparing intralesional 5-fluorouracil.

1. 5-FU is first drawn into a 1-mL syringe to the first graduated line. Each graduated line represents a volume of 2/100 mL and will contain 1 mg of 5-FU.
2. Triamcinolone is then added to 5-FU in the same syringe by drawing 2/10 mL of Kenalog-10, which contains 2 mg of triamcinolone.
3. Normal saline is then drawn into the same syringe to a total volume of 1.0 mL.

While very low doses of the chemotherapy drugs mentioned above pose no risks to adult patients, pharmaceuticals that are genotoxic and target rapidly dividing cells are assumed to be teratogenic and/or lethal to an embryo or fetus.

In female patients, the growth and maturation phase of folliculogenesis (4–6 months) is most susceptible to persisting DNA damage, which can potentially result in embryo–fetal malformations.

In male patients, the chemotherapy drugs may cause DNA damage in sperm, potentially resulting in adverse effects on the conceptus with a female sexual partner.

To minimize the risk of adverse embryo–fetal effects, all patients—male or female—shall be warned about the potential embryo–fetal risks of ILC and advised to use contraception during the course of treatment and for a period of 6 months after cessation of therapy.

### Calcium Channel Blocker

Verapamil is a calcium channel blocker that has been studied for the topical and intralesional treatment of keloids. In vitro studies of verapamil have demonstrated antifibrotic activity, inhibition of collagen synthesis, increased secretion of collagenase, decreased production of IL-6 and vascular endothelial growth factor (VEGF), reduced cell proliferation, and increased apoptosis. 20 A double-blind randomized controlled trial with a paired split-scar design, comparing intralesional verapamil and ILT, was terminated early because it appeared that verapamil was not as effective as ILT in preventing keloid recurrence after excision. 20 However, a single-blinded randomized controlled trial with 32 patients reported that the triamcinolone–verapamil blend is as effective as ILT monotherapy in reducing Vancouver scar scale scores of postsurgical keloids in a shorter time frame and with fewer side effects. 21

In a recent randomized controlled trial, the combination of verapamil and triamcinolone provides a more effective treatment for keloids, thereby highlighting the potential of verapamil in scar reduction. Another recent study revealed that TAC was more effective than verapamil for improving vascularity; TAC was superior to verapamil in improving height within 9 weeks of treatment; TAC produced superior results for improving pliability within 18 weeks of treatment, whereas verapamil produced superior results between 18 and 24 weeks of treatment. Verapamil had fewer adverse events than TAC and can be used as a safer alternative for treating keloids. 22 However, this needs further validation. 21

### Botulinum Toxin A

Botulinum toxin type A (BTA) is a powerful neurotoxin produced by the gram-positive bacterium *Clostridium botulinum* , causing temporary muscle paralysis that lasts for 2 to 6 months. 23 Increased skin tension may explain why keloids occur more frequently in certain locations, 24 and the reduction of mechanical forces by BTA could be a mechanism that inhibits keloid formation and growth. BTA directly affects dermal cells, such as fibroblasts and keratinocytes, and can positively mediate dermal tissue remodeling through them. BTA appears to disrupt the differentiation of fibroblasts into myofibroblasts by blocking TGF-β1 signaling, thereby reducing excessive wound retraction and scarring thickening. 25

A recent meta-analysis indicated that treatment with BTA combined with ILT leads to a significant improvement in visual analog scale scores and Vancouver Scar Scale scores, compared with control groups receiving either ILT or BTA alone. 26 However, the study also notes that no statistically significant difference in scar thickness was found among the groups.

### Laser Therapy

Similar to surgical therapy, laser therapy should only be used as an adjunct to other treatments, such as intralesional corticosteroids. The lasers employed for treating keloids can be classified into ablative lasers, non-ablative lasers, and fractional lasers. Ablative lasers include the 2,940-nm Er:YAG laser and the 10,600-nm CO _2_ laser. These lasers ablate tissue by targeting water molecules within it, which serve as a major absorbing chromophore. Furthermore, they induce coagulation and thermal damage in the surrounding tissue, which may trigger keloid formation as a side effect. The extent of thermal damage tends to be greater with CO _2_ lasers compared with Er:YAG lasers. Non-ablative lasers for keloids include the long-pulsed 1,064-nm Nd:YAG laser and the 585-nm and 595-nm pulsed dye lasers. Their mechanism of action may involve the destruction (coagulation) of capillaries by the absorption of laser light energy by hemoglobin. Additionally, direct anti-inflammatory effects have been suggested, along with the creation of edema within a few minutes, which could facilitate the intralesional application of drugs. To enhance the penetration of topical agents into the dermis, LADD using fractional ablative lasers may be considered. This method creates microchannels in the skin, allowing for improved delivery of topical treatments for keloids.

According to a Cochrane review of 2022 including 15 RCTs with 604 participants, there is currently insufficient evidence to support or refute the effectiveness of different laser therapies for keloids and hypertrophic scars. 27

### Oral Tranilast

Tranilast, an antiallergy drug, is a derivative of tryptophan and has been studied for its role in scleroderma, keloid and hypertrophic scars, liver fibrosis, renal fibrosis, cardiac fibrosis, pulmonary fibrosis, and uterine fibroids by inhibiting TGF-β1, may reduce subjective symptoms by suppressing scar inflammation. 28 29

Tranilast exerts antifibrotic effects by suppressing fibrotic pathways, including TGF-β and mitogen-activated protein kinase. Because it disrupts fibrotic pathways and has demonstrated beneficial effects against keloid and hypertrophic scars, tranilast could be used to treat other conditions characterized by fibrosis. 30

### Compression Therapy

Compression therapy can be administered through various methods, including pressure dressings and elastic bandages. 31 It may be used alongside other treatments, particularly surgical interventions, to help prevent keloid recurrence. The exact mechanism of this treatment remains not fully understood. In hypertrophic scars, studies have shown that compression decreases the cohesiveness of collagen fibers 31 and encourages collagen degradation. 32 Compression therapy is frequently applied to earlobe keloids, where its application is more manageable than in other areas. While it is generally well-tolerated, inconsistency in wearing compression devices may hinder the effectiveness of the therapy.

A meta-analysis conducted in 2009 found that pressure garment therapy significantly reduced scar height. 33 It significantly reduced the Vancouver Scar Scale score, scar thickness, redness, pigmentation, and hardness in another meta-analysis in 2017. 34

#### Silicone Gel Sheeting

Silicone gel sheeting has a nearly 40-year history, including widespread clinical use for 20 years. The beneficial effects of silicone sheets may reflect their ability to stabilize and moisturize the scar, thus decreasing inflammation. 35 36 It is postulated that applying silicone gel to an acute scar with a deficient epithelial water barrier due to an immature stratum corneum results in decreased water loss.

A 2021 Cochrane review of 13 studies with 468 participants concluded that there is limited evidence from high-quality trials about the clinical efficacy of silicone sheeting in the treatment of hypertrophic scars. 37

#### Corticosteroid Tape

With minimal overlap with normal skin, the tape should be applied to the scar. It should initially be used continuously for at least 3 months, changing every 24 to 48 hours. Flurandrenolide tape 4 μg/cm ^2^ , a high-potency topical corticosteroid, is available in the United States, Brazil, the United Kingdom, and several European countries. Plasters containing deprodone propionate are available only in Japan, where these tapes have served as a first-line therapy for keloids and hypertrophic scars. Despite its limited availability in many countries, the postoperative application of corticosteroid tapes and plasters significantly helps prevent keloid recurrence.

### Scar Massage

Scar massages are used to enhance skin qualities such as pliability, adhesions, pruritus, and pain. There is little evidence supporting the benefits of this approach in reducing hypertrophic scars. 38 39 It is essential for massage therapy to align with the scar maturation stage. The inflammation of the scar is the primary consideration factor for this treatment.

### Surgery

“The simpler the approach is, the better we can expect from the surgery for keloids.”

**Fig. 2:**
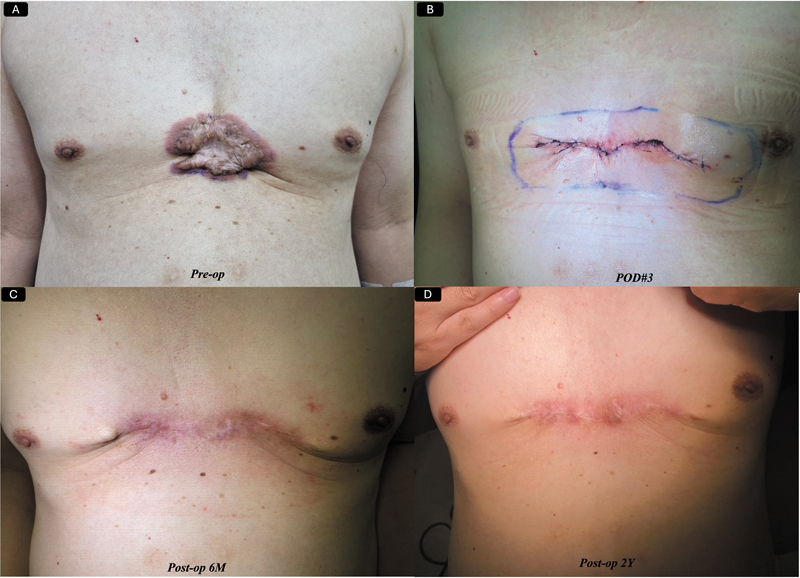
( **A** ) A 61-year-old male patient presented to our clinic with severely painful anterior chest wall keloids. We performed complete excision with primary closure followed by a single-fraction radiotherapy of 10.5 Gy on the day of surgery. ( **B** ) The appearance at postoperative day 3, ( **C** ) the appearance at 6 months postoperatively, and ( **D** ) the 2-year follow-up results.

**Fig. 3:**
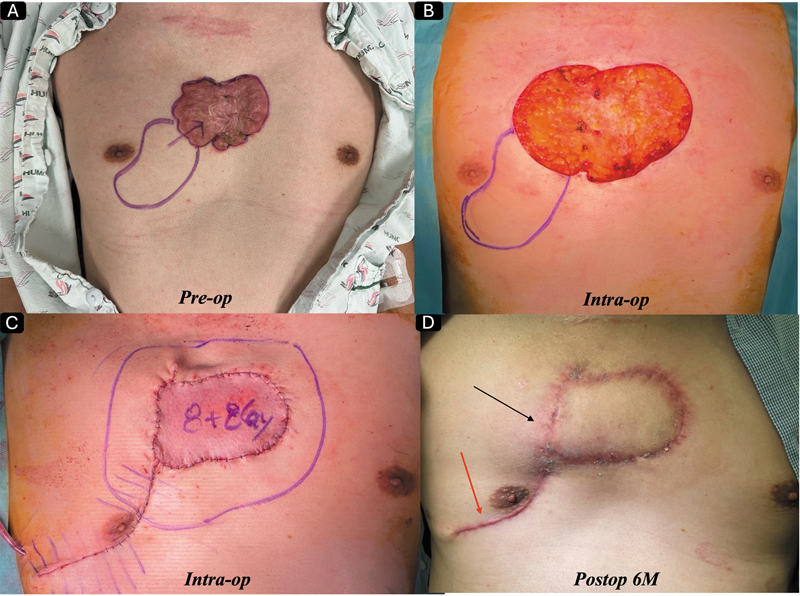
( **A** ) A 50-year-old male patient visited our clinic with severely painful anterior chest wall keloids. We completely excised the lesion and covered the defect with a perforator flap, followed by two fractions of 16 Gy (8 Gy at postoperative day 0 and 8 Gy at postoperative day 1). For safety reasons, we spared the nipple -areolar complex area from the radiotherapy field. As shown, a mild keloid recurrence occurred postoperatively in the lesion that did not receive radiotherapy; however, this recurrence can be safely managed with intermittent corticosteroid injections. The patient has been recurrence-free for 1 year after treatment (the last picture taken was at 6 months post-op, as seen in Fig D ).

Numerous attempts and studies have been conducted to improve surgical therapy for keloid patients. Nevertheless, the keloid recurrence following surgery has been reported to range from less than 5% to up to 100%. This significant variation is likely a reflection of a lack of standardization of surgical therapy for keloid disorder. From what we have observed, surgical therapy for this entity should balance patients' desires and what surgeons can realistically provide, considering potential success rates and possible complications. This is the key to success and should be thoroughly discussed with patients before initiating surgery. Generally, the surgical aspect of keloids has two themes. “How do you remove it and close it thereafter?” The surgery for keloid removal can be categorized into complete excision versus partial excision (or intralesional excision). 40 Complete excision seems to have a better prognosis once it is done correctly. As surgeons, we learned the “reconstructive ladder,” and keloid disorder is a very good example of how we should abide by this custom, no matter what. In this regard, whenever possible, primary closure after complete keloid excision would be ideal in most cases. Complete keloid excision followed by primary closure is planned and attempted when indicated, mainly dependent on tissue laxity, anatomical location, keloid orientation, and multiplicity in that specific region. For example, in a sizable chest keloid, complete excision followed by primary closure is recommended when the keloid lesion is less than 6 cm in height in a resting position, 41 as shown in Fig. 2 . If the keloids are out of that range, flap closure should be considered, which can be finally decided intraoperatively after complete excision, as shown in Fig. 3 .

The problem arises when patients have multilevel keloids in tension-prone areas. In these cases, we can proceed with excision in multiple stages. Typically, unless the multilevel lesions are amenable to simultaneous excision at a time, more symptomatic lesions can be excised entirely first, while the remaining lesions can be addressed later in a future operation.

**Fig. 4:**
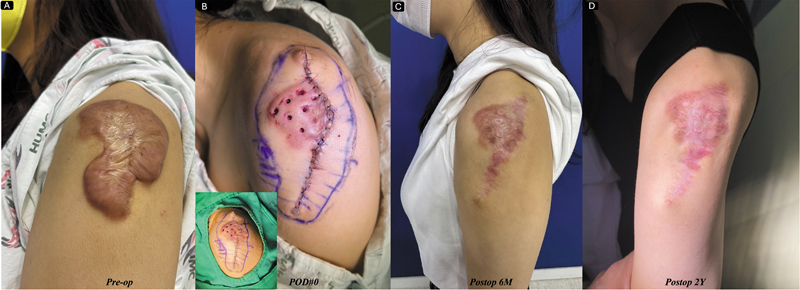
( **A** ) A 34-year-old woman visited our clinic with severely itchy, painful keloids on her left upper arm, which developed after BCG vaccination in her youth. ( **B** ) We performed a combination of complete and partial punch excisions, followed by 11-Gy single-fraction radiotherapy on the day of surgery. ( **C** ) For the lesion treated with punch excision, we injected corticosteroids twice. ( **D** ) She has remained free of recurrence for 2 years after treatment.

**Fig. 5:**
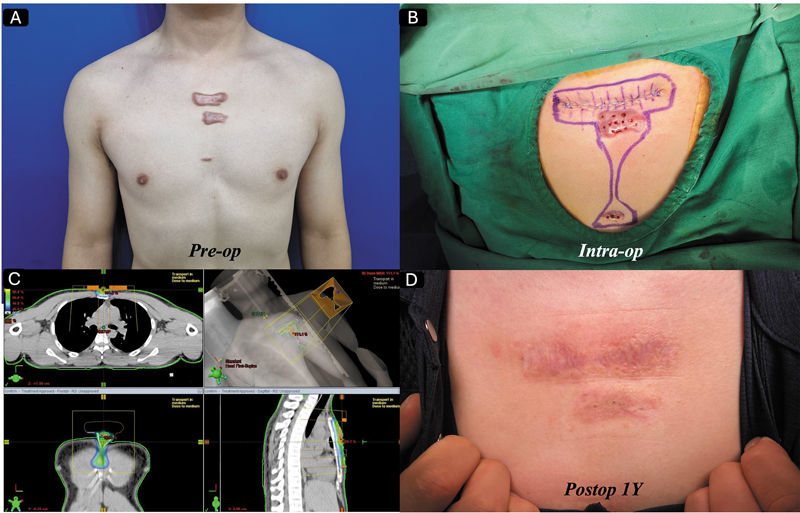
( **A** ) A 34-year-old woman visited our clinic with severely itchy, painful keloids on her left upper arm, which developed after BCG vaccination in her youth. We performed a combination of complete and partial punch excisions, followed by 11-Gy single-fraction radiotherapy on the day of surgery. ( **C** ) For the lesion treated with punch excision, we injected corticosteroids twice. ( **D** ) She has remained free of recurrence for 2 years after treatment.

Some authors recently introduced punch excision in keloid surgery, which remains one of the most significant advances in the surgical management of keloids. 40 42 This technique is most attractive because if the lesion is relatively large, we can combine complete and punch excision on the same lesion. The representative cases are shown in Figs. 4 and 5 . The most fascinating fact of this approach is that it can be extremely effective in massive multiple keloid cases that have been considered incurable for many, as we published in 2025.

**Fig. 6:**
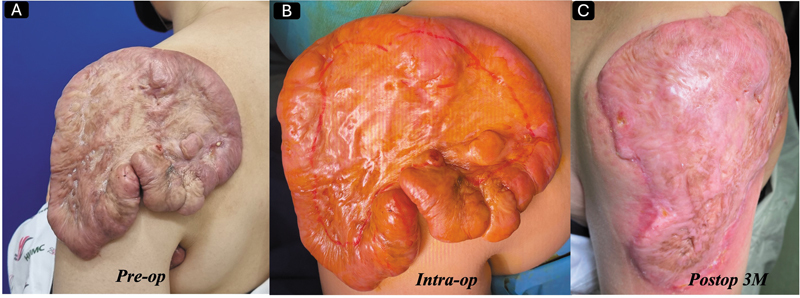
( **A** ) A 48-year-old female patient came to our clinic with severely itching, painful keloids on her left shoulder of unknown cause. ( **B** ) We performed a marginal debulking surgery (outlined in red) followed by two sessions of 16 Gy (8 Gy at postoperative day 0 and 8 Gy at postoperative day 1). ( **C** ) She has been free from recurrence for 18 months after treatment, with the last picture taken at 3 months post-op.

If the lesion has extreme pain or itchiness, the keloid should be adequately addressed, especially in extremely massive keloids. We recommend marginal debulking surgery along with appropriate radiotherapy when indicated, as shown in Fig. 6 .

This flexible, patient-oriented surgical strategy is critical for successful treatment, particularly in tension-prone anatomical areas. It is especially useful for symptom relief and a better quality of life for genetically susceptible patients with multiple massive keloids. 42

Surgery alone may be associated with an extremely high recurrence rate (up to 100%) despite proper techniques, such as reducing tension on the dermis, using subcutaneous/fascial tension-reducing sutures, and/or performing local flap transfers. Flaps are superior to skin grafts because the latter do not expand postsurgery and may lead to higher recurrence rates during long-term follow-ups. 43

**Fig. 7:**
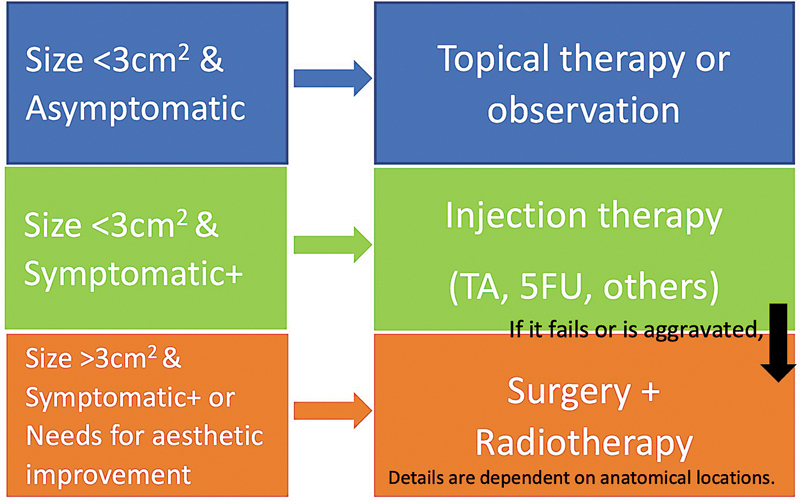
The flowchart for primary keloid surgical treatment for tension-prone site keloids (except for the ear). TA, triamcinolone acetonide; 5-FU, 5-fluorouracil.

Although anatomical locations determine the appropriate surgical approach, we recommend following the flowchart for keloid treatment, except for the ear ( Fig. 7 ).

### Postoperative Radiation Therapy

In adult patients, radiation therapy is crucial after keloid surgery because it minimizes the formation of blood vessels and reduces inflammation in the incisional wound. 44 While the radiation source was previously superficial or orthovoltage X-rays (photons) and it is still adopted as a main therapy in some institutions, 45 an electron beam (β-ray) is now often preferred because it exposes internal organs to less radiation. 46 47 High-dose-rate brachytherapy may also be a viable option; however, its long-term safety is yet to be established. 48 Radiation therapy, however, is generally contraindicated in the pediatric population.

#### Recent Updates on Postoperative Radiotherapy

Single-fraction electron beam radiotherapy (EBRT) with a 10-Gy dose has emerged as a highly effective and safe adjuvant treatment for preventing keloid recurrence following surgical excision.

Studies of single-fraction protocols (10 Gy in a single fraction) report recurrence rates ranging from 0 to 16%, often matching or outperforming multifraction schemes. High single doses can target fibroblasts with low α/β ratios more effectively, generating greater DNA damage and reactive oxygen species, critical mechanisms in preventing recurrence. A one-time session avoids repeated hospital trips, reducing patient burden, unlike conventional three to five fractions. Especially for patients with limited access to radiation centers, the simplicity of a single session improves adherence. As seen in bone metastasis protocols, single-fraction is more cost-effective—around 25 to 30% cheaper—without diminishing quality-adjusted life years. 49

Han et al 50 (2024) performed a retrospective analysis of 48 patients with 71 earlobe keloids treated with surgical excision followed by immediate postoperative single-fraction 10-Gy EBRT within 24 hours. They achieved a 100% recurrence-free rate with minimal side effects (two cases of grade III radiation dermatitis). Ha et al 51 (2023) performed a prospective study on anterior chest keloids treated with surgical excision followed by immediate postoperative single-fraction 10-Gy EBRT. No recurrences were observed during the follow-up period.

Kang et al 47 (2024) performed a comparative analysis between 9.5 and 10 Gy single-fraction EBRT administered within 24 hours postsurgery to 182 patients with ear keloids. The 10-Gy group had a significantly lower recurrence rate (0.81%) than the 9.5-Gy group (8.47%). Radiation timing within 24 hours did not significantly affect recurrence rates. They concluded that 10-Gy single-fraction EBRT is the most effective low-dose treatment for preventing ear keloid recurrence.

Park 52 (2023) performed a retrospective analysis of their experience treating keloids with complete excision and omega variant keystone flap, followed by postoperative single-fraction 10-Gy EBRT for helical keloids. They included 21 patients, 25 helical keloids (May 2021–March 2023), and no keloid recurrence across all 25 lesions, with a mean follow-up of 12 months.

Sruthi et al 53 (2014) performed a retrospective study on 37 keloid lesions treated with surgical excision followed by single-fraction EBRT (5–12 Gy) within 24 to 72 hours. The 5-year recurrence-free rate was 73.4% for those receiving 8 Gy.

Single-fraction EBRT (10 Gy × 1) administered within 24 to 72 hours postexcision is a clinically effective, patient-friendly, cost-effective adjuvant strategy that may offer radiobiological superiority over traditional multifraction regimens in preventing keloid recurrence. However, based on T.H.P.'s unpublished experience, high-tension areas such as the anterior chest wall or the shoulder could be considered cautiously between 1 to 2 and 3 to 5 fractions.

Table 1 shows the comparison between multiple- and single-fraction radiotherapy, and Table 2 shows the outcome of various studies published.

**Table 1:** Comparison of single versus multifraction radiotherapy for keloids

Parameter	Single fraction	Multifraction
Toxicity	Low rates of ≥grade II reactions	Comparable or slightly higher acute toxicity
Patient visits	1	3–5
Cost	25–30% cheaper per case	Higher direct and indirect costs
Compliance	Excellent (minimal burden)	Lower in populations with access limitations
Radiobiology	Enhanced fibroblast kill and DNA damage	Lower per-fraction biologic effect

**Table 2:** Overview of the outcome of electron beam radiotherapy

First author (year)	Number of keloids	Dose (Gy × fx)	Biologically effective dose2 (Gy ^2^ )	Recurrence rate (%)	Location
Mitsuhashi (1995) 61	42	5 **×** 3	52.5	26.2	Mixed
Mitsuhashi (1995) 61	20	6 **×** 3	72	20	Mixed
Ogawa (2003) 62	147	5 **×** 3	52.5	32.7	Mixed (including ear)
Heianna (2004) 63	29	4 **×** 3	36	20.7	Ear
Ogawa (2007) 64	284	5 **×** 3	52.5	28.2	Mixed
Ogawa (2007) 64	28	5 **×** 2	35	0	Ear
Ogawa (2007) 64	58	5 **×** 4	70	17.2	Mixed
Yossi (2013) 65	112	3 **×** 5	37.5	31.3	Not specified
Ogawa (2013) 66	47	5 **×** 3	52.5	4.3	Ear
Ogawa (2013) 66	127	5 **×** 2	35	3.9	Ear
Wang (2014) 67	54	5 **×** 4	70	9.3	Mixed (including ear)
Wang (2014) 67	60	4 **×** 5	60	33.3	Mixed
Aluko-Olokun (2014) 68	53	4 **×** 4	48	41.5	Face (some ear)
Capel (2015) 69	17	3 **×** 5	37.5	23.5	Ear
Ogawa (2015) 70	63	5 **×** 3	52.5	4.8	Ear
Kim (2015) 71	16	5 **×** 3	52.5	0	Ear
Carvajal (2016) 72	103	5 **×** 3	52.5	35	Not specified
Bennett (2017) 73	31	4 **×** 3	36	74.2	Ear
Khalid (2018) 74	30	5 **×** 2	35	56.7	Ear
Renz (2018) 75	125	5 **×** 4	70	1.6	Not specified
Renz (2018) 75	45	4 **×** 4	48	8.9	Not specified
Renz (2018) 75	80	3 **×** 4	30	10	Not specified
Liu (2018) 76	15	5 **×** 4	70	13.3	Chest
Liu and Yuan (2019) 76	30	5 **×** 3	52.5	13.3	Ear
Arima (2019) 77	141	6 **×** 3	72	10.6	Anterior chest wall
Petrou (2019) 78	16	5 **×** 3	52.5	6.3	Not specified
Dohi (2019) 79	38	6 **×** 3	72	5.3	Upper arm
Maemoto (2020) 80	61	5 **×** 3	52.5	29.5	Mixed
Maemoto (2020) 80	13	5 **×** 4	70	23.1	Mixed
Dohi (2020) 81	34	7.5 **×** 2	71.3	8.8	Umbilicus
Lin (2020) 82	43	4.5 **×** 3	43.9	14	Mixed
Wen (2021)	151	4 **×** 5	60	15.2	Ear
Hwang (2022) 83	119	6 **×** 3	72	6.7	Ear
Hwang (2022) 83	17	5 **×** 3	52.5	11.8	Ear
Ha (2023) 84	16	10 **×** 1	60	18.5	Anterior chest wall
Han (2024) 85	71	10 **×** 1	60	0	Earlobe
Kang (2025) 85	182	9.5 **×** 1 or 10 **×** 1	54.6 and 60	8.47 and 0.81	Ear

#### Primary Radiation Therapy

While radiation therapy is less effective than surgery combined with postoperative radiotherapy, it has also been used as a standalone treatment to address keloids. 54 Generally, the total radiation dose is higher than that used in postoperative radiation.

### Regenerative Medicine Therapies

A 2024 systematic review assessed the efficacy and safety of regenerative treatments for hypertrophic scars and keloids. 55 The study highlighted that platelet-rich plasma, stromal vascular fraction, and stem cell-conditioned medium have demonstrated effectiveness with minimal side effects. These therapies can be used alone or in combination with standard treatments, offering a promising approach for keloid management. They concluded that human adipose-derived stem cells-derived extracellular vesicles play a significant role in preventing hypertrophic scar formation, potentially offering a novel therapeutic approach for managing excessive scarring during wound healing. Their ability to influence matrix remodeling and cytokine regulation positions them as promising candidates for future clinical applications in wound healing and scar management.

### Electrical Stimulation Therapy

Electrical stimulation has recently emerged as a novel therapeutic modality for scar modulation. A July 2024 scoping review ( *Advances in Wound Care* ) discusses potential benefits of extracorporeal shockwave therapy for keloid pain and appearance, though clinical data are preliminary. 56 Kang et al reported that low-frequency, low-intensity alternating currents have been shown to suppress collagen production and enhance matrix remodeling by altering fibroblast membrane dynamics. 57 This approach offers a promising, non-invasive strategy for treating keloids and hypertrophic scars.

### Targeted Molecular Therapies

Researchers at the University of Cincinnati identified CYP24A1 as a new molecular target for treating keloids. Inhibiting CYP24A1 with compounds like ketoconazole and VID400 reduced the expression of profibrotic genes and keratinocyte proliferation in keloid tissue. 58 This discovery opens avenues for targeted therapies in keloid management. Selective inhibition of prolyl-tRNA synthetase by DWN12088 represents a novel molecular approach to suppress collagen synthesis in keloids, offering promising preclinical efficacy and a potential path toward first-in-class antifibrotic therapy. 59

### Laser-Assisted Drug Delivery

LADD is an emerging technique that enhances the penetration of therapeutic agents into the skin. Combining a fractional CO _2_ laser with topical corticosteroids for keloids has shown improved outcomes compared with traditional methods. 60 This approach may offer more effective treatment options for keloid scars. However, these studies are limited in sample size and lack long-term follow-up outcomes. Larger randomized controlled trials with a wide variety of topical drugs are required to validate LADD's efficacy and side effects before this technique can be employed as a standard of treatment.

## Conclusion

Multimodal, individualized treatment approaches—guided by lesion morphology, anatomical location, treatment history, and patient factors—are essential for optimizing outcomes. First-line therapies, including intralesional corticosteroids and cryotherapy, continue to play a central role, while ILC offers a viable option for refractory lesions. Surgical excision, when followed by timely postoperative radiation therapy—especially single-fraction EBRT—has shown promising efficacy, particularly in auricular keloids.

Emerging therapies such as BTA, calcium channel blockers, LADD, and regenerative medicine are expanding the therapeutic arsenal, offering additional strategies for resistant or recurrent cases. Moreover, the integration of molecular and genetic insights is paving the way for the development of targeted therapies, which may ultimately transform keloid treatment into a more precise and effective discipline.

Moving forward, well-designed, large-scale clinical trials and international consensus guidelines are urgently needed to validate current practices and establish robust, evidence-based treatment algorithms. Until then, clinicians must rely on patient-centered, flexible strategies that incorporate both traditional and emerging modalities to improve outcomes and quality of life for those affected by this burdensome condition.
